# Case report: Novel GJB2 variant c.113T>C associated with autosomal recessive non-syndromic hearing loss (ARNSHL) in a Han family

**DOI:** 10.1097/MD.0000000000018253

**Published:** 2019-12-16

**Authors:** Xinqiang Lan, Shiyu Sun, Xin Lan, Linyuan Niu, Chunxiao Zhang, Xiaoli Chen, Ningning Xia

**Affiliations:** a Department of Medical Genetics, Weihai Maternity and Child Care Hospital; b Department of Medical Genetics, Weihai Municipal Second Hospital Affiliated to Qingdao University, Weihai, Shandong Province; c Medical College, Nanchang University, Nanchang, Jiangxi Province, China.

**Keywords:** autosomal recessive non-syndromic hearing loss, gene microarray, gap junction beta 2 protein gene, Sanger sequencing

## Abstract

**Rationale:**

Molecular mechanism underlying the autosomal recessive non-syndromic hearing loss (ARNSHL) is still plausible. Pathogenic mutations of the gap junction beta 2 protein (GJB2) are reported to be the primary causes of ARNSHL.

**Patient concerns:**

A propositus was diagnosed as ARNSHL with bilateral congenital profound hearing loss.

**Diagnosis:**

With microarray and target gene sequencing testing methods, a novel GJB2 mutant was found to be associated with ARNSHL in this Han Chinese family.

**Interventions/Outcomes:**

Based on the finding in this research, prenatal screening of GJB2 mutation and genetic counseling are recommended to this family for their next pregnancy. Our interventions allow the family to plan informatively.

**Lessons:**

In this family, we discovered 2 heterozygous carriers of c.113T>C variation in the GJB2 gene. The propositus, who had profound hearing loss, had inherited the c.113T>C variation from his normal mother and the c.235delC from his father.

## Abstract 2

Supplemental Digital Content is available in the text

## 1 Introduction

Hearing loss is one of the most congenital forms of sensory impairment, with an incidence of approximately 2.5 in 1000 births worldwide.^[1]^ It is estimated that approximately 60% of all sensorineural hearing loss cases has a genetic cause; >70% of these genetic cases can classified as nonsyndromic hearing loss (NSHL), in which hearing impairment is the only obvious clinical abnormality.^[2]^ One of the most common inherited hearing loss types is autosomal recessive nonsyndromic hearing loss (ARNSHL), which accounts for approximately 80% of all cases.^[2,3]^ Molecular mechanisms underlying this symptom are still under investigation and approximately 100 hearing loss causing genes have been identified, and over 200 gene loci have been mapped (http://heretidaryhearingloss.org/).

The gap junction beta 2 protein (*GJB2*) gene, which encodes for the gap junction protein connexin 26, is playing important roles in molecular pathways associated ARNSHL.^[4–6]^ In the previous study, GJB2 mutations such as *GJB2* c.235delC and c.299–300delAT are commonly identified in Asian ARNSHL patients.^[7,8]^ However, around 30% of rare *GJB2* gene mutation has been shown pathogenicity in ARNSHL patients. Thus, the discovery of *GJB2* variants associated with ARNSHL will enhance our understanding of ARNSHL and improve the efficiency of early diagnosis. The diverse ethnicities, coupled with the high mutation frequencies among ethnic groups, studying certain ethnic group are important. In this study, we focus on study the GJB2 mutation spectrum in a Han family; some of the family members have ARNSHL.

## 2 Methods

The Committee of Genetics and Reproductive Ethics at the Weihai Maternity and Child Care Hospital approved this study (WMCCH-2017–56). The experiment and research were conducted with the written informed consent of all participants. The methods were performed in accordance with the approved guidelines.

This study was conducted on a Chinese family with 6 members, 3 of whom were patients of ARNSHL. Participants were screened in the Weihai Maternity and Child Care Hospital. The propositus was diagnosed with bilateral congenital profound hearing loss in December 2015 at her first visit to our hospital. The clinical findings of the family members with hearing loss, tinnitus, vestibular symptoms, and other clinical abnormalities were reviewed. Through follow-up work, the propositus became pregnant and chose to give birth to her child in September 2016. The audiometric evaluations included use of the auditory brainstem responses (ABR), speech recognition. Pure tone audiometry (PTA) for air conditions at 0.25, 0.5, 1, 2, 4, and 8 kHz was used to calculate the average of the hearing threshold for the better-hearing ear of each affected subject. The subjects were evaluated using ABR. The severity of their hearing impairments was defined as mild (26–40 dB HL), moderate (41–70 dB HL), severe (71–90 dB HL), or profound (> 90 dB HL). We can access to information that could identify participants during or after data collection.

Genomic DNA was extracted from whole blood samples using a blood DNA kit, according to the standard protocol (TIANGEN BIOTECH, Beijing, China), and a total 2 μL DNA sample was added to detect quantity and purity with an ultraviolet spectrophotometer.

Deafness gene microarray testing was used to investigate the *GJB2* c.235delC, c.299–300delAT, c.35delG, and c.176del16 pathogenic variants; *GJB3* c.538C>T, *SLC26A4* c.2168A>G, and c.IVS7-2A>G pathogenic variants; and mitochondrial *12S rRNA* c.1555A>G and c.1494C>T pathogenic variants using the 9 hereditary deafness gene detection array kits (CapitaBio, Beijing, China). The standard procedures were used to detect 9 pathogenic variants in the 4 genes, as recommended by the manufacturer. Hybridization reactions were performed using a BioMixerTM II Microarray Hybridization Station (CapitaBio, Beijing, China), and a LuxScanTM 10K Microarray Scanner (CapitaBio, Beijing, China) was employed for microarray data capture.

Polymerase chain reaction (PCR) and Sanger sequencing were utilized to detect and determine the propositus’ genetic variants; they were then used on her family members except for her husband; this was performed to study potential mutation in the causative gene. The *GJB2* coding region sequence was investigated through NCBI GenBank, and by using Primer 5.0 software (London, Ontario, Canada) to design the primers of the *GJB2* gene. The following primers were designed to amplify Exons 1 and 2 of *GJB2*: *GJB2*-ex1-Forward, 5′-CGGGGGAGACTCAGGGC-3′; *GJB2*-ex1-Reverse, 5′-AGGGACATCGGCGACACC-3′; *GJB2*-ex2-Forward, 5′-CTGTTTTGGTGAGGTTG-3′; *GJB2*-ex2-Reverse and 5′-CACATTGTCCATAGACTGAT-3′. PCR amplification was executed under the following conditions: 94 °C for 30 seconds, 32 cycles of 94 °C for 30 seconds, 62 °C for 30 seconds, 72 °C for 30 seconds, and 72 °C for 10 minutes. The purified PCR products were sent for Sanger sequencing (Sangon Biotech, Shanghai, China). The sequencing results were then analyzed using Chromas software and aligned against the *GJB2* (NG_008358.1) sequence shown in the NCBI database.

Bioinformatics predictive tools including Mutationtaster, PolyPhen, SIFT, and Provean to assess possible effects of mutations on the protein. Database including: the single nucleotide polymorphism database (dbSNP), the National Heart Lung and Blood Institute exome sequencing project, and the 1000 genome project were employed for evaluation purpose.

## 3 Case report

**Figure 1:**
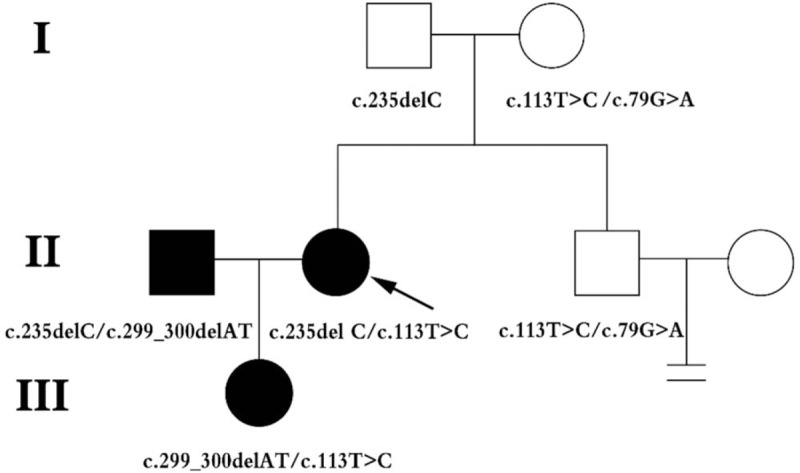
Pedigree shows the segregation of the c.113T>C missense mutation with the syndrome. This pedigree shows the members of 2 families are affected with NSHL. The genotype of affected members is shown in the figure. NSHL = nonsyndromic hearing loss.

**Figure 2:**
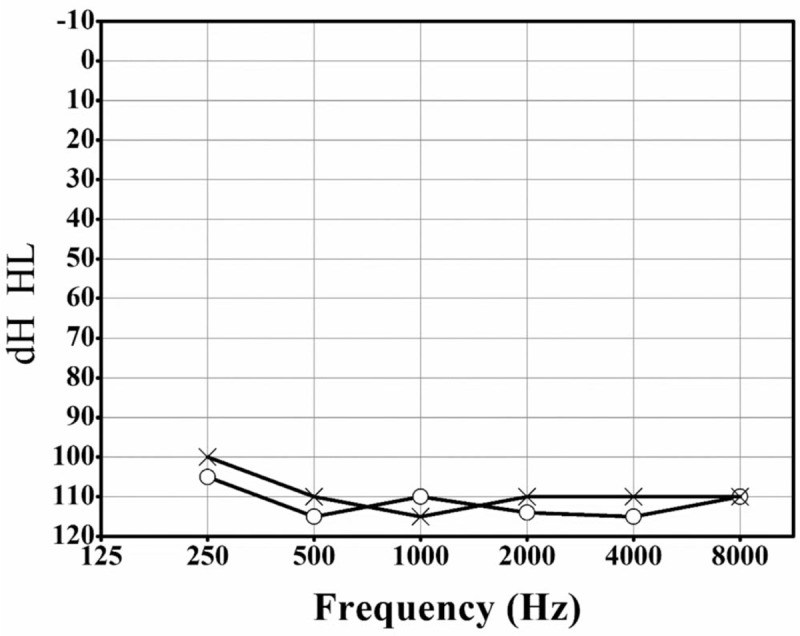
Audiograms of both ears from the propositus. Symbols “o” and “x” denote conduction pure-tone thresholds at different frequencies in the right and left ear, respectively.

**Figure 3:**
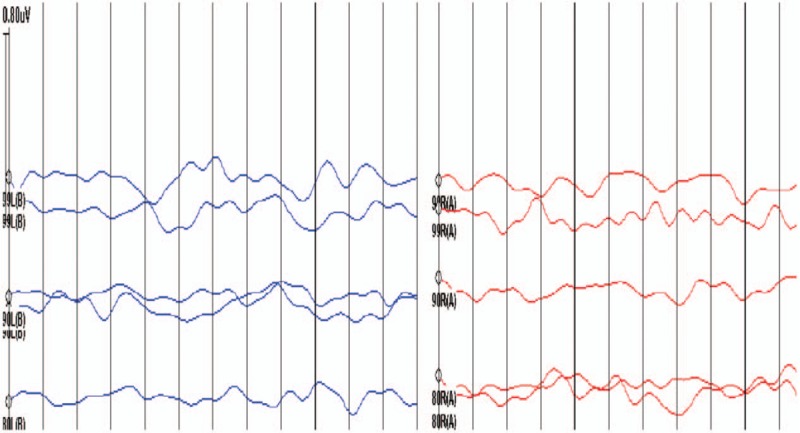
ABR of both ears from the child of the propositus. (A) Right ear of the child; (B) left ear of the child.

The study sample consisted of 6 family members, including 3 clinically affected and 3 unaffected individuals (Fig. 1). The propositus (II: 2) was a 28-year-old woman diagnosed with bilateral congenital profound hearing loss in 2015 at her first visit to our hospital. The hearing impairment result of the propositus was in Fig. 2. Through follow-up work, the propositus became pregnant and chose to give birth to her child (III: 1). Seventy hours postpartum, III: 1's Transient OtoAcoustic Emissions (TrOAEs) were observed to be absent at all frequencies; at 3 months, the child's ABR could not be evoked in either ear (Fig. 3).

**Figure 4:**
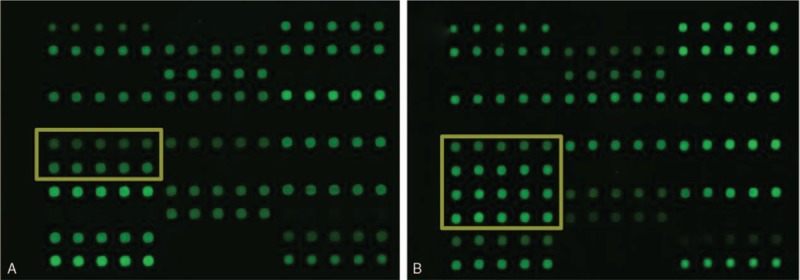
Microarray analysis for deafness gene mutations. Panel (A) denotes the result of propositus shows that the single heterozygous c.235delC mutation of *GJB2* gene; Panel (B) denotes the result of II:1 shows that compound heterozygous c.235delC/ c.299-300delAT mutations of *GJB2* gene.

The propositus’ deafness gene microarray results demonstrate a single heterozygous c.235delC pathogenic variant in the *GJB2* gene's (Fig. 4A). II: 1's microarray reveals compound heterozygous c.235delC/c.299-300delAT pathogenic variants (Fig. 4B). Many researchers have identified the *GJB2* c.235delC mutation as the most prevalent, while c.299-300delAT mutation are considered as the second most common mutations found in the Chinese population.^[4]^ Thus the cause for II: 1's auditory impairment was identified by the deafness gene microarray.

**Figure 5:**
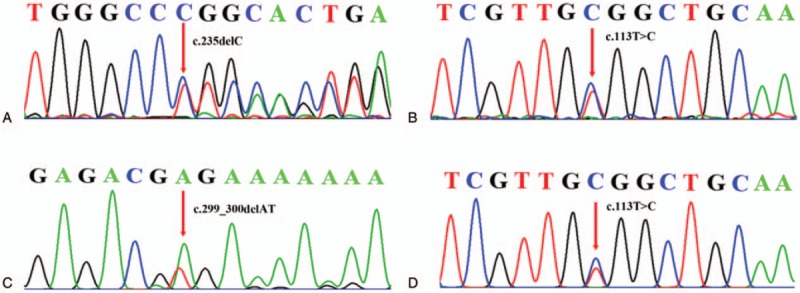
Sequencing graph shows the new compound heterozygous of *GJB2*. (A) c.235delC mutation and (B) c.113T>C mutation of propositus (II: 2); (C) c.299-300delAT and (D) c.113T>C mutation of her child (III: 1).

Sanger sequencing was used to further study the causes for the propositus’ pathogenesis (Fig. 5A and B). The results of the Sanger sequencing confirmed compound heterozygous c.235delC/ c.113T>C mutations of her *GJB2* genes. In order to investigate the meaning of the c.113T>C mutation, Sanger sequencing was employed to detect I: 1, I: 2, and II: 3. In S1 Fig, the Sanger sequencing results indicated I: 1 as being the single heterozygous c.235delC pathogenic variant of the GJB2 gene; moreover, I: 2 and II: 3 were found to be compound heterozygous 79G>A/c.113T>C mutations. In addition, Sanger sequencing result of III: 1 revealed compound heterozygous c.299-300delAT/c.113T>C mutations in the *GJB2* gene (Fig. 5C and D). Based on the above results, a c.113T>C mutation in the *GJB2* gene was found as a novel candidate gene locus for hearing loss.

## 4 Discussion

**Figure 6:**
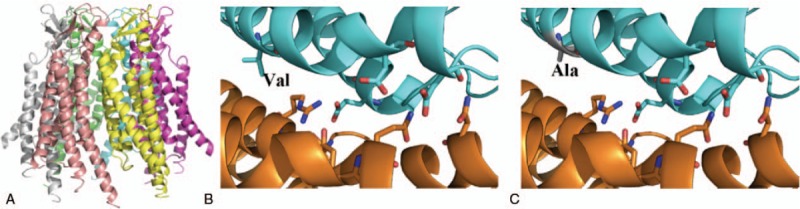
With the well-annotated 3D structure of *GJB2* protein. (A) 3D structure of *GJB2* protein; (B) p. Val of 38 protein; (C) the c.113T>C (p. Val38Ala).

To examine the molecular causes of ARNSHL diagnosed in 3 members of a Han family, microarray assay and Sanger sequencing are employed to study the mutants associated with the phenotypes. Based on the clinical phenotype and gene microarray results, the propositus’ case may involve an unusual pathogenesis (Figs. 2 and 4). From the Sanger sequencing results illustrated in Fig. 5, the propositus clearly possesses compound heterozygous c.235delC/c.113T>C mutations which are inherited the c.113T>C variation from her normal mother and the c.235delC from her father. The observation of no hearing loss in her mother indicates that a single heterozygous c.113T>C mutation of the *GJB2* gene would not cause the hearing loss (Fig. 2). It is been reported that c.113T>C (p. V38A) is a missense mutation, affecting protein function through non-synonymous substitutions of connexin 26.^[11]^ These lead us to deduce that the c.113T>C mutation of the *GJB2* gene might be a novel candidate mutation for autosomal recessive non-syndromic hearing loss. Bioinformatics evaluation scores of *GJB2* c.113T>C supports our hypothesis that V38AGJB2 could be an important candidate mutant affecting the structure and function of GJB2. The 3D structure of the *GJB2* protein, (http://www.ebi.ac.uk/pdbe/entry/pdb/5ER7) (Fig. 6), shows that Valine 38 localizes in the C-terminal of helix structures through contact with multiple amino acid residues.^[12]^

The connexin 26 gene, identified by Kelsell et al^[6]^ in 1997, was the first to be associated with nonsyndromic sensorineural autosomal deafness. Although mutations in the *GJB3* and *GJB6* genes were subsequently discovered, *GJB2* remains the most commonly known cause of hereditary hearing loss in many population.^[9,10,13]^*GJB2*, located on human chromosome 13q11,^[6,14]^ encodes the connexin 26 (CX26) protein, a member of the connexin family of highly related gap junction proteins. Connexins oligomerize to form connexons, present in membrane proteins, which can bind with connexons from adjacent cells to form functional gap junctions.^[14,15]^ Numerous varieties of deafness-causing mutations of the *GJB2* gene are extremely common, while others are quite rare. The c.235delC and c.299-300delAT, reported as being more prevalent in the most Asian, are caused by frameshift mutations resulting from base deletions, which lead to a shift in reading the frame of the codon just after the mutated site. This results in a premature termination of the protein's translation and the formation of a non-functional truncated connexin 26 gap junction protein, leading to deafness.^[16–18]^ Nonetheless, the pathogenicity of missense mutations depends on many factors, such as the location of the mutation in the protein and the nature of the substitution.^[14]^ The finding of missense mutations involving the location of p. Val38Ala in the domain of a propositus has never been reported.

Our results demonstrate that a c.113T>C recessive mutation of the *GJB2* gene plays a key role in identifying the candidate gene for ARNSHL. The present findings may be used in the development of prenatal diagnoses and genetic counseling, and have an important implication for greatly reducing the birth rate of deaf children and improving the quality of births.

## 5 Conclusions

This is the first report expressing the candidacy of c.113T>C as being causative in the *GJB2* gene mutations associated with a risk in ARNSHL development. We believe that identifying more of these variations is essential to precise molecular diagnosis and the decision to obtain genetic counseling, and to better guiding family planning for the improved management of heritable deafness.

## Acknowledgments

The authors wish to thank all of the propositus’ family members for their participation and cooperation in this study.

## Author contributions

C**onceptualization:** Xinqiang Lan, Linyuan Niu, Xin Lan, Chunxiao Zhang, Xiaoli Chen, Ningning Xia.

**Data curation:** Xin Lan, Linyuan Niu.

**Formal analysis:** Xiaoli Chen.

**Methodology:** Xin Lan, Ningning Xia.

**Project administration:** Xiaoli Chen, Ningning Xia.

**Software:** Shiyu Sun Xiaoli, Chen, Ningning Xia.

**Supervision:** Ningning Xia.

**Validation:** Chunxiao Zhang.

**Visualization:** Chunxiao Zhang.

**Writing – original draft:** Shiyu Sun, Chunxiao Zhang, Xiaoli Chen.

**Writing – review & editing:** Xinqiang Lan, Shiyu Sun

## Supplementary Material

Supplemental Digital Content
