# Supplementary material for: Case report: Novel GJB2 variant c.113T>C associated with autosomal recessive non-syndromic hearing loss (ARNSHL) in a Han family
Source: Medicine (Baltimore). 2019 Dec 16;98(50):e18253. doi: 10.1097/MD.0000000000018253 (PMC6922571; doi:10.1097/MD.0000000000018253)

**Supporting information**

**S1 Fig.**  **Sequencing graph shows the new compound heterozygous of *GJB2*.** (A) c.235delC mutation of I: 1; (B) 79G>A mutation and (C) c.113T>C mutation of I: 2; (D) 79G>A mutation and (E) c.113T>C mutation of II: 3.


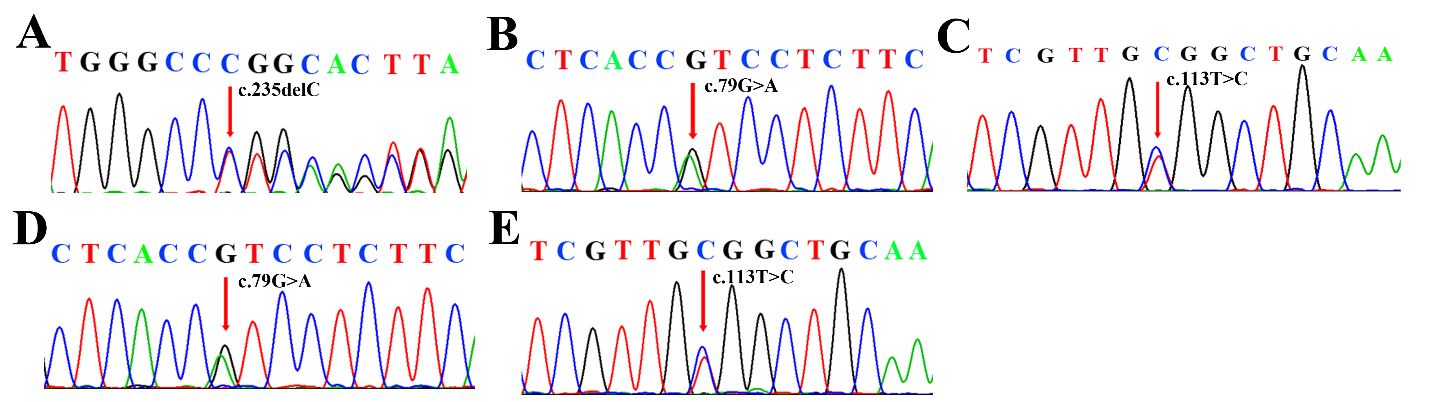

Supplement: Supplemental Digital Content [file medi-98-e18253-s001.docx]
